# Prevention of tooth extraction-triggered bisphosphonate-related osteonecrosis of the jaws with basic fibroblast growth factor: An experimental study in rats

**DOI:** 10.1371/journal.pone.0211928

**Published:** 2019-02-08

**Authors:** Mitsuhiko Imada, Takahiro Yagyuu, Yoshihiro Ueyama, Masahiko Maeda, Kazuhiko Yamamoto, Satoshi Kurokawa, Jun-ichiro Jo, Yasuhiko Tabata, Yasuhito Tanaka, Tadaaki Kirita

**Affiliations:** 1 Department of Oral and Maxillofacial Surgery, Nara Medical University, Kashihara-shi, Nara, Japan; 2 Laboratory of Biomaterials, Department of Regeneration Science and Engineering, Institute for Frontier Life and Medical Sciences, Kyoto University, Sakyo-ku, Kyoto, Japan; 3 Department of Orthopedic Surgery, Nara Medical University, Kashihara-shi, Nara, Japan; Nanjing Medical University, CHINA

## Abstract

Osteonecrosis of the jaw induced by administration of bisphosphonates (BPs), BP-related osteonecrosis (BRONJ), typically develops after tooth extraction and is medically challenging. As BPs inhibit oral mucosal cell growth, we hypothesized that suppression of the wound healing-inhibiting effects could prevent BRONJ onset after tooth extraction. Since basic fibroblast growth factor (bFGF) promotes wound healing, but has a short half-life, we examined whether the initiation of BRONJ could be prevented by applying a bFGF-containing gelatin hydrogel over the extraction sockets of BRONJ model rats. Forty-three rats, received two intravenous injections of zoledronic acid 60 μg/kg, once per week for a period of 2 weeks, underwent extraction of a unilateral lower first molar. The rats here were randomly assigned to the bFGF group (n = 15 rats, gelatin hydrogel sheets with incorporated bFGF applied over the sockets); the phosphate-buffered saline (PBS) group (n = 14 rats, gelatin hydrogel sheets without bFGF applied over the sockets); or the control group (n = 14 rats, nothing applied over the sockets). One rat in the bFGF group was sacrificed immediately after tooth extraction. Twenty-one rats were sacrificed at 3 weeks, and the remaining 21 rats were sacrificed at 8 weeks after tooth extractions. The harvested mandibles were analyzed using micro-computed tomography and sections were evaluated qualitatively for mucosal disruption and osteonecrosis. The incidence of osteonecrosis at 8 weeks after tooth extraction was 0% in the bFGF group, 100% in the PBS group, and 85.7% in the control group. The frequency of complete coverage of the extraction socket by mucosal tissue was significantly greater in the bFGF group than in the other groups. These results suggest that application of bFGF in the extraction socket promoted socket healing, which prevented BRONJ development. The growth-stimulating effects of bFGF may have offset the inhibition of wound healing by BP.

## Introduction

Bisphosphonates (BPs) that inhibit bone resorption are frequently used to treat osteoporosis, skeletal-related events, multiple myeloma, Paget’s bone disease, and other conditions that result in bone fragility [1–3]. However, osteonecrosis of the jaw induced by administration of BPs, BP-related osteonecrosis (BRONJ), has become a serious problem.

The severity of BRONJ is classified into four stages on the basis of clinical and radiographic findings, and stage-specific treatment strategies have been proposed (Table 1). Nevertheless, once established, BRONJ is often refractory to treatment and tends to be progressive. In a cohort study characterizing BRONJ among patients receiving intravenous BPs over a median follow-up period of 1.5 years, 25%, 48%, and 27% of patients receiving stage-specific treatment improved, stayed within the same stage, and worsened, respectively [4]. Progression of BRONJ in terms of severity causes pathologic fractures, oral antral/oral nasal communication, extra-oral fistulas, and so on, resulting not only in a marked decrease in quality of life [5], but also sometimes in a fatal outcome due to sepsis [6]. At present, no standard of treatment for BRONJ has been established; thus, it is important to prevent the onset of BRONJ.

**Table 1:** Staging and treatment strategies for BRONJ.

Stage	Description	Treatment strategies
0	Non-specific clinical findings, radiographic findings and symptoms. No clinical evidence of necrotic bone.	Systemic management. Use of analgesics and antibiotics.
1	Asymptomatic, but exposed and necrotic bone or fistulas are present. No evidence of infection.	Use of chlorhexidine mouthrinse, close monitoring of patient on quarterly basis. Provide patient education.
2	Exposed necrotic bone or fistulas that probes to bone in area of infection. Clinical signs of infection, such as pain, erythema with or without purulent discharge.	Symptomatic treatment with antibiotics, oral bacterial mouthrinse, pain control, debridement of infected area.
3	Exposed necrotic bone or fistulas present that probes to bone and extends beyond region of alveolar bone, such as to inferior border of mandible, ramus of mandible, and zygoma in maxilla. Fracture of jaw or osteolysis present in jaws.	All of the above treatment, plus more aggressive surgical intervention, such as debridement and resection of jaw.

Taken from Ruggiero et al. [3]

The pathogenesis of BP-induced BRONJ is thought to involve inhibition of osteoclastic bone resorption and remodeling, inhibition of angiogenesis, soft tissue toxicity, immune dysfunction, etc. [3]. It has been reported that 63.7% of BRONJ cases are associated with tooth extraction [7]; hence, the influence of BPs on bone remodeling has been actively studied. However, the effects have not yet been clarified, and no prophylaxis has been established.

In this study, we focused on the effects of BPs on soft tissue toxicity. Recent basic research has revealed that BPs inhibit the growth of oral mucosal cells [8]. Therefore, we hypothesized that suppression of the inhibitory effects of BPs on wound healing could prevent the initiation of BRONJ after tooth extraction. We speculated that the application of basic fibroblast growth factor (bFGF), which promotes wound healing [9], over the tooth extraction sockets, could prevent the initiation of BRONJ after tooth extraction. However, bFGF has an extremely short half-life in vivo (more than 80% of the bFGF injected subcutaneously was eliminated from the injected site within 24 hours [10]). We therefore focused on gelatin hydrogel as a representative drug delivery system [11]. This carrier allows controlled release of incorporated growth factors over a long period of time [12–14]. Hence, in this study, we examined whether the onset of BRONJ could be prevented by applying gelatin hydrogel containing bFGF over the extraction sockets of rats administered BPs.

## Materials and methods

### Preparation of gelatin hydrogel sheets

Gelatin with an isoelectric point of 5.0 was supplied by Nitta Gelatin (Osaka, Japan), and human recombinant bFGF with an isoelectric point of 9.6 was supplied by Kaken Pharmaceutical (Tokyo, Japan). Gelatin hydrogel sheets were made as previously described [15] through chemical crosslinking of aqueous gelatin solution with 25% glutaraldehyde (Nacalai Tesque, Inc., Kyoto, Japan). Briefly, polytetrafluoroethylene molds were filled with 5% aqueous gelatin solution containing glutaraldehyde. Crosslinking was allowed to proceed at 4°C for 12 hours, and the resultant hydrogel was immersed in 100 mM glycine aqueous solution at 37°C for 1 hour to block the residual aldehyde groups of the glutaraldehyde. Thereafter, the gelatin hydrogel was freeze-dried and gelatin hydrogel sheets with 95% water content were produced.

To prepare the gelatin hydrogel sheets incorporating bFGF, 100 μg of bFGF was diluted with 200 μl of PBS (Gibco, Life Technologies, Roskilde, Denmark). Next, bFGF was incorporated into the gelatin hydrogel sheet by dropping 200 μl of bFGF solution onto a freeze-dried gelatin hydrogel sheet (20 mg, 11 mm × 13 mm, 1.0-mm thick), which was then left at 37°C for 1 hour. Gelatin hydrogel sheets without bFGF were prepared by dropping 200 μl of PBS onto a freeze-dried gelatin hydrogel sheet (20 mg, 11×13 mm, 1.0 mm thick), which were then left at 37°C for 1 hour.

### Creation of a rat BRONJ model

This animal study was approved by the animal care and use committee of Nara Medical University (Permit Number: 11756). Forty-three female Sprague-Dawley (SD) rats (Japan SLC, Shizuoka, Japan), aged 10 weeks, were used in this study. The rats were kept in an environment with a controlled temperature and 12-hour light–dark cycles, with food and water supplied *ad libitum*. Surgery was performed under general anesthesia and all efforts were made to minimize suffering.

**Fig 1:**
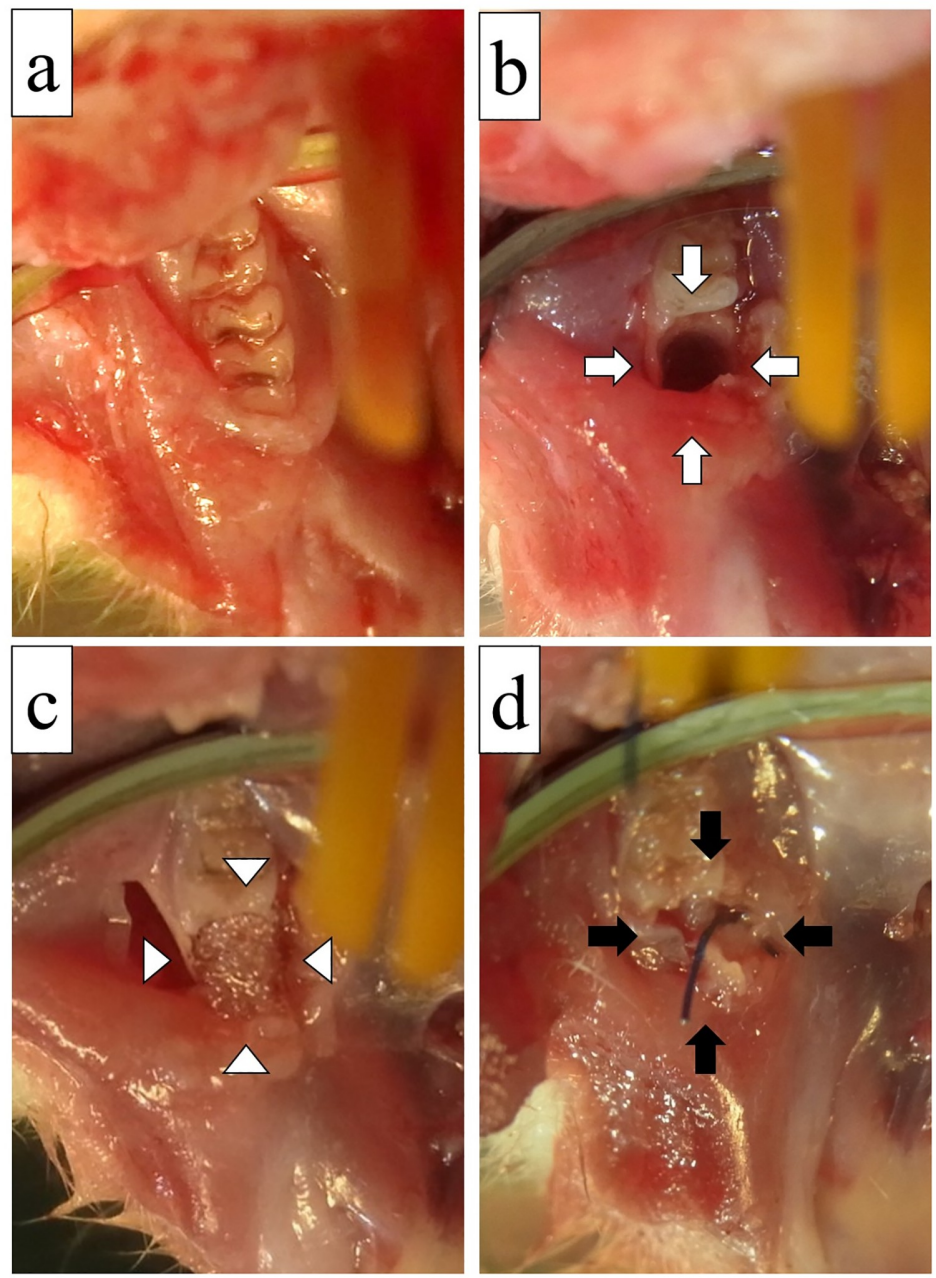
Application of a gelatin hydrogel sheet incorporating bFGF. (a) The lower first molar before extraction. (b) After tooth extraction, the extraction socket was expanded with a twist drill to create a bone defect (open arrow). (c) The alveolar defect after applying a gelatin hydrogel sheet (open arrowhead). (d) The bone defect was sutured primarily (filled arrow).

We conducted this study using a rat BRONJ model, similar to the model used in a previous study by Borke et al. [16]. All rats received two intravenous injections of zoledronic acid (ZA) 60 μg/kg based on the human dosage of 4 mg/65.8 Kg, once per week for a period of 2 weeks. The second dose was administered on the date of the molar extraction, 1 week after the first dose. The intravenous ZA injections were administered into the tail vein, after application of a rubber band tourniquet placed at the base of the tail to reveal the vein. After induction of anesthesia with a single intraperitoneal injection of pentobarbital sodium 50 mg/kg, extraction of the unilateral lower first molar was performed in each rat (Fig 1). The previous study [16] and our own preliminary experiments (S1 Fig) showed that none of the non-ZA-treated rats exhibited osteonecrosis.

A gingival sulcus incision was made around the lower first molar using a #11 surgical blade. A mucoperiosteal flap was raised using a periosteal elevator. The molars were removed using a sharpened dental explorer. A 2-mm-diameter twist drill was used to expand the extraction sockets to a standardized depth of 2 mm. Each extraction socket was expanded with a drill to create a standardized bone defect with a size of 2 × 2 × 3 mm. The rats were randomly assigned to one of three groups, as follows: the bFGF group (n = 15 rats), where gelatin hydrogel sheets with bFGF incorporated were applied over the alveolar defects; the phosphate-buffered saline (PBS) group (n = 14 rats), where gelatin hydrogel sheets without bFGF were applied over the defects; and the control group (n = 14 rats), where nothing was applied over the defects. The prepared gelatin hydrogel sheet with or without bFGF (for the bFGF and PBS groups), or nothing (for the control group) was placed in the defect. Finally, the bone defect was sutured by placing a horizontal mattress suture using 5/0 polypropylene. One rat in the bFGF group was sacrificed immediately after tooth extraction for comparison with time. Twenty-one rats were sacrificed at 3 weeks after tooth extractions, and the remaining 21 rats were sacrificed at 8 weeks after tooth extractions. Rats were sacrificed by exsanguination under general anesthesia.

### Micro-computed tomography (CT) analysis

The harvested mandibles were analyzed using a micro-CT system (Toscaner-32300 μ-FPD; Toshiba IT and Control Systems, Tokyo, Japan). Each mandible was scanned at intervals of 38 μm at 100 kV and 200 μA. Three-dimensional images were constructed using VG Studio software (Volume Graphics, Heidelberg, Germany).

**Fig 2:**
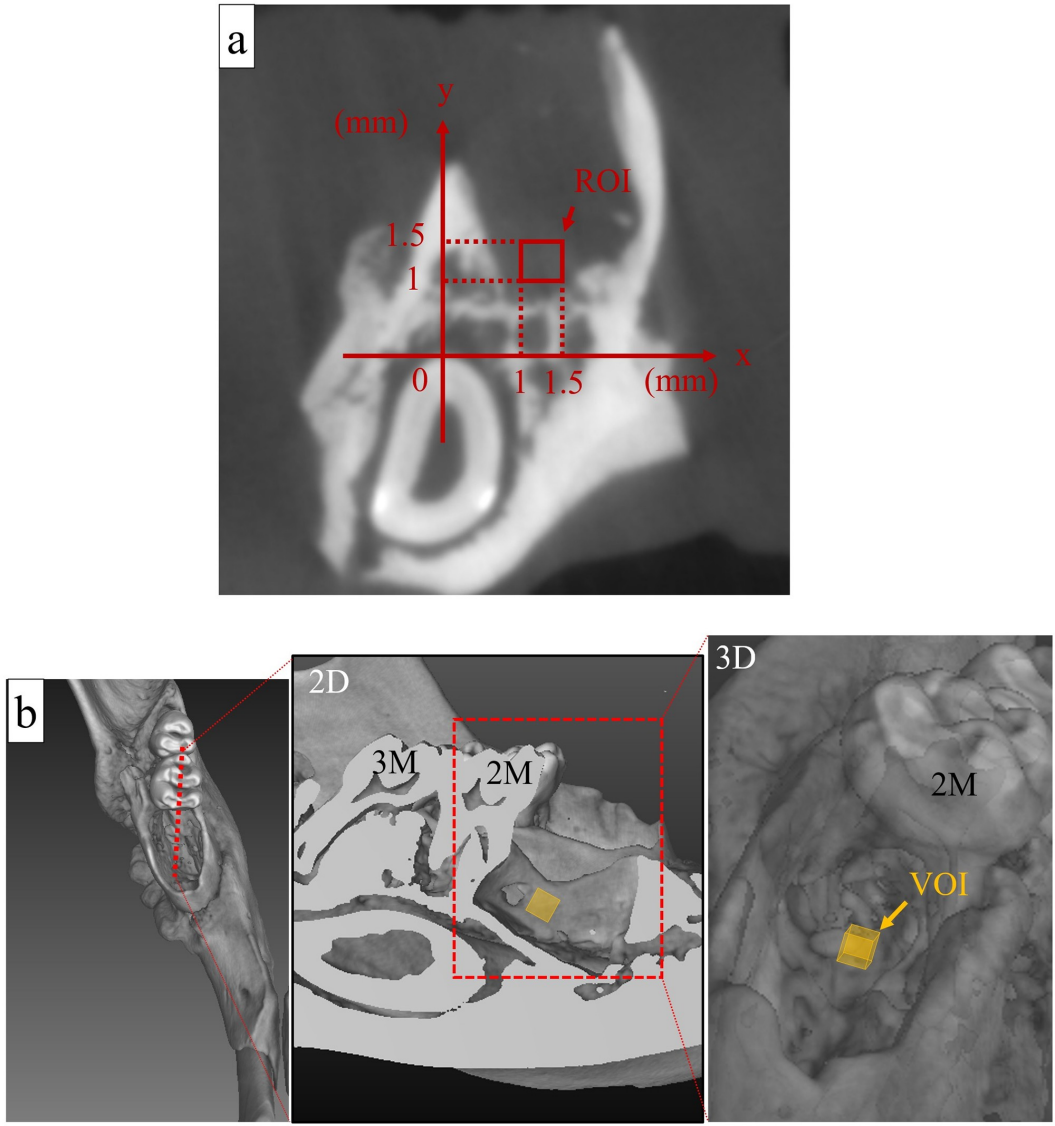
Setting of the region of interest (ROI) and the volume of interest (VOI) in the alveolar defect. (a) The ROI was set at 1.0-mm mesial from the mesial surface of the lower second molar on the CT coronal section. The tangent line passing through the highest point of the incisor, parallel to the occlusal plane, was set as the x-axis, and the perpendicular line passing through that point was set as the y-axis. The highest point of the incisor was set as the origin, and a square region of 0.5 mm × 0.5 mm with vertices at (x, y) = (1 mm, 1 mm), (1 mm, 1.5 mm), (1.5 mm, 1 mm), (1.5 mm, 1 mm), and (1.5 mm, 1.5 mm) was defined as the ROI. (b) By auto-interpolation between layers, ROI became the volume of interest (VOI) in the area between the coronal sections 1.0- and 1.5-mm mesial from the mesial surface of the lower second molar. The volumes of the newly formed bone in this VOI were determined 3 and 8 weeks after tooth extraction, and compared among the study groups. (2D, two-dimensional view; 3D, three-dimensional view; 2 M, lower second molar; 3 M, lower third molar).

The finding in each alveolar defect was assessed on a coronal section 1.0-mm mesial from the mesial surface of the lower second molar. Additionally, to examine the newly formed bone in the defect 3 or 8 weeks after tooth extractions, we measured the calcification areas (which were high-density areas, and defined as having a density equal to or greater than 150 CT units [17]), within the region of interest (ROI) in the socket (Fig 2a). By auto-interpolation between layers, ROI became the volume of interest (VOI) in the area between the coronal section 1.0- and 1.5-mm mesial from the mesial surface of the lower second molar (Fig 2b). VOI was determined to be 0.125 mm^3^, and the volume of the newly formed bone was calculated within the VOI. We used ImageJ software (v. 1.49; NIH, Bethesda, MD, USA) for the analysis.

### Histological analysis

After micro-CT analysis, all mandibles were fixed in 10% formaldehyde neutral buffer solution, decalcified with 0.5 mol/l EDTA, and embedded in paraffin. Thin sections, 5 μm in thickness, were cut at 1.0-mm mesial from the mesial surface of the lower second molar in the buccolingual direction and stained with hematoxylin and eosin (H&E) for a light microscopic examination. These sections were evaluated qualitatively for the presence or absence of mucosal disruption and osteonecrosis. Osteonecrosis was defined as exposed bone persistent at 8 weeks postoperatively. Moreover, the total number of empty osteocyte lacunae per extraction socket were determined 3 and 8 weeks after tooth extraction, and compared among the study groups. Investigators, blinded to the results of the micro-CT analyses and to the identification of the rat groups, independently performed the histological analysis.

### Statistical analysis

To assess the significance of differences in the volumes of newly formed bone, as determined by micro-CT analysis, and the total number of empty osteocyte lacunae per extraction socket, as determined by histological analysis, statistical analysis was performed using the Mann-Whitney U test. The incidence of alveolar defects with exposed bone based on macroscopic findings and the presence or absence of mucosal disruption and osteonecrosis determined by histological analysis were evaluated using Steel’s test. Differences with P values of less than 0.05 were considered significant for both tests.

## Results

### Changes in body weight

The mean body weight of the rats was 215.0 g (SE 2.1 g) at the beginning of ZA administration and 227.6 g (SE 2.4 g) after tooth extraction. In addition, the mean body weights of rats at the time of sacrifice 3 and 8 weeks after tooth extraction were 243.3 g (SE 3.5 g) and 278.1 g (SE 6.3 g), respectively. Thus, regardless of the treatment after tooth extraction, the body weight increased in all rats.

### Macroscopic findings

**Fig 3:**
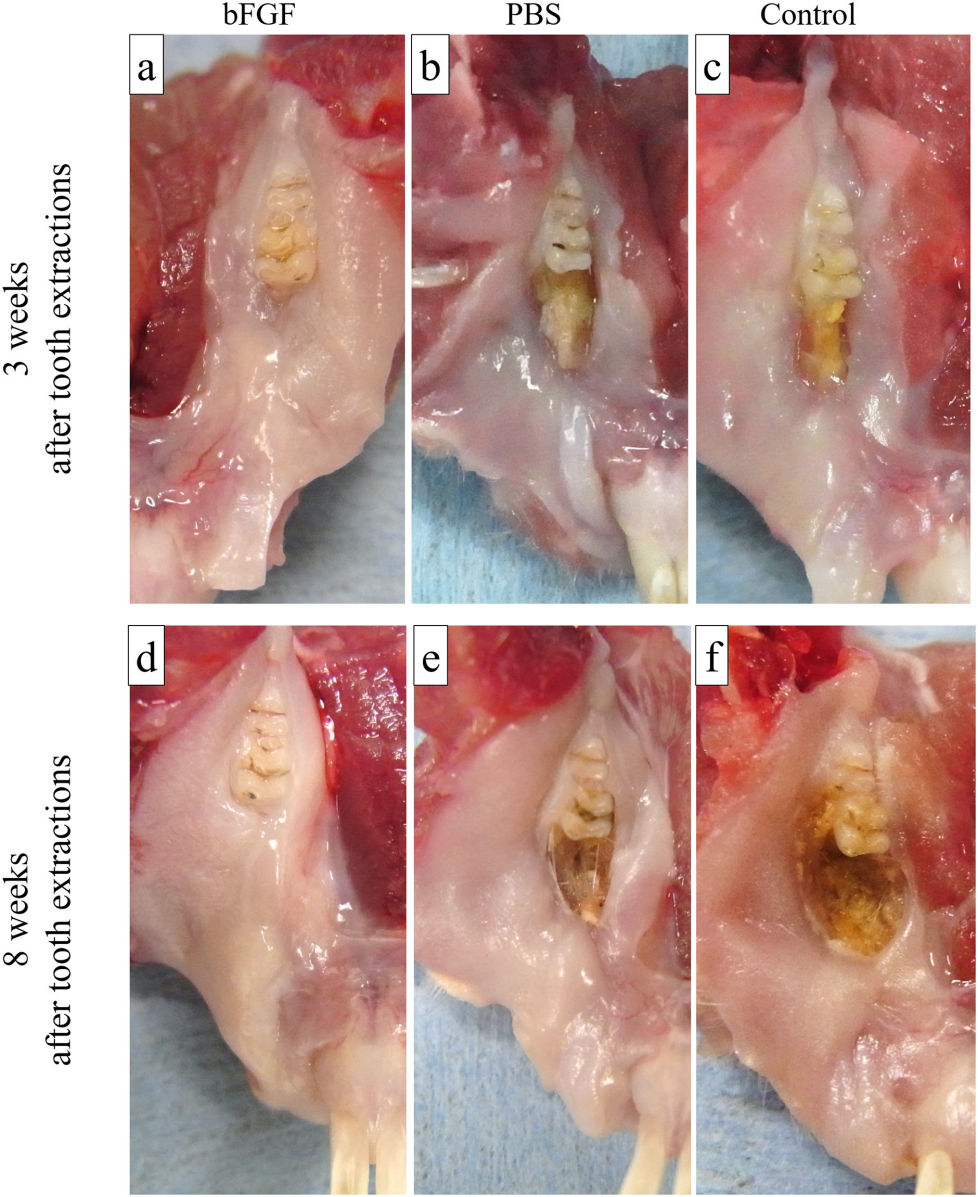
Typical macroscopic findings. (a) Extraction socket in the bFGF group at 3 weeks after tooth extraction. (b) Extraction socket in the PBS group at 3 weeks after tooth extraction. (c) Extraction socket in the control group at 3 weeks after tooth extraction. (d) Extraction socket in the bFGF group at 8 weeks after tooth extraction. (e) Extraction socket in the PBS group at 8 weeks after tooth extraction. (f) Extraction socket in the control group at 8 weeks after tooth extraction. The extraction sockets in the bFGF group at 3 and 8 weeks (a, d) after the tooth extraction socket was covered with normal mucosa, similar to the surrounding areas, and demonstrating no signs of inflammation. The concavities of the sockets were still present at 3 weeks after tooth extraction (a), but there was no residual socket concavity at 8 weeks after tooth extraction (d). The extraction sockets in the PBS group (b) and the control group (c) at 3 weeks after tooth extraction showed exposed bone of normal color. The extraction sockets in the PBS group (e) and the control group (f) at 8 weeks after tooth extraction showed discolored, brownish exposed bone.

Three weeks after tooth extraction, the extraction socket in the bFGF group (Fig 3a) was covered with normal mucosa, similar to the surrounding areas, demonstrating no signs of inflammation, such as redness; however, the concavities of the sockets were still present. The extraction sockets in the PBS group (Fig 3b) and the control group (Fig 3c) showed exposed bone of normal color and without pus discharge.

Eight weeks after tooth extraction, the extraction socket in the bFGF group (Fig 3d) showed no inflammatory changes, and was covered with normal mucosa. Additionally, there were no residual socket concavities. The extraction sockets in the PBS group (Fig 3e) and the control group (Fig 3f) showed discolored, brownish exposed bone, sometimes with accompanying pus discharge.

The incidence of osteonecrosis based on the macroscopic finding of exposed necrotic bone at 3 and 8 weeks after tooth extraction was evaluated (Table 2). The incidence of osteonecrosis in the bFGF group, the PBS group, and the control group at 3 weeks after tooth extraction was 14.3% (1/7), 71.4% (5/7), and 85.7% (6/7), respectively. The incidence of osteonecrosis in the control group was significantly higher than in the bFGF group. The incidence of osteonecrosis in the bFGF group, the PBS group, and the control group at 8 weeks after tooth extraction were 0% (0/7), 57.1% (4/7), and 57.1% (4/7), respectively. The incidence of osteonecrosis in the PBS group and the control group were significantly higher than in the bFGF group.

**Table 2:** The incidence of extraction sockets with exposed bone based on macroscopic findings.

	bFGF	PBS	Control
3 weeks after tooth extraction	1/7 (14.3%)	5/7 (71.4%)	6/7* (85.7%)
8 weeks after tooth extraction	0/7 (0%)	4/7* (57.1%)	4/7* (57.1%)

* P<0.05 vs. bFGF

### Micro-CT findings

**Fig 4:**
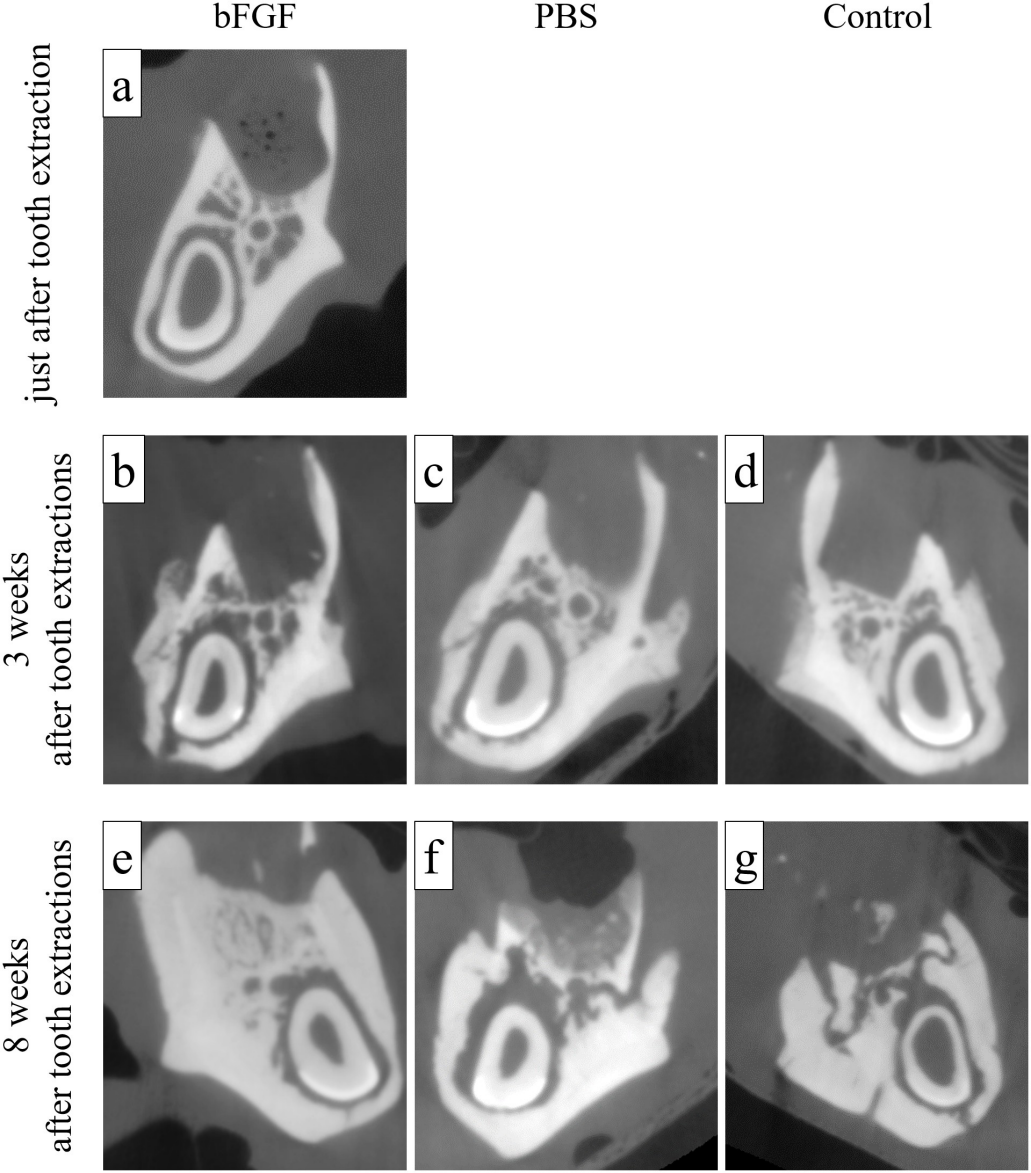
Typical micro-CT images. (a) Alveolar defect in the bFGF group immediately after tooth extraction. (b) Alveolar defect in the bFGF group at 3 weeks after tooth extraction. (c) Alveolar defect in the PBS group at 3 weeks after tooth extraction. (d) Alveolar defect in the control group at 3 weeks after tooth extraction. (e) Alveolar defect in the bFGF group at 8 weeks after tooth extraction. (f) Alveolar defect in the PBS group at 8 weeks after tooth extraction. (g) Alveolar defect in the control group at 8 weeks after tooth extraction. There was no bone formation in the alveolar defects in any group at 3 weeks after tooth extraction (b–d). Whereas bone formation was found in the bFGF group (e) at 8 weeks after tooth extraction, no such bone formation was found in the PBS group (f) and the control group (g), but small bone fragments suggestive of bone sequestra were present at this time point.

Compared to the micro-CT images obtained immediately after tooth extraction (Fig 4a), the images obtained at 3 weeks after extraction revealed no marked difference in the morphology of alveolar defects between each group (Fig 4b–4d). Micro-CT images obtained 8 weeks after tooth extraction showed bone formation in the extraction sockets in the bFGF group (Fig 4e). On the other hand, small bone fragments suggestive of sequestra were found without evident bone formation in the PBS group (Fig 4f) and the control group (Fig 4g).

**Fig 5:**
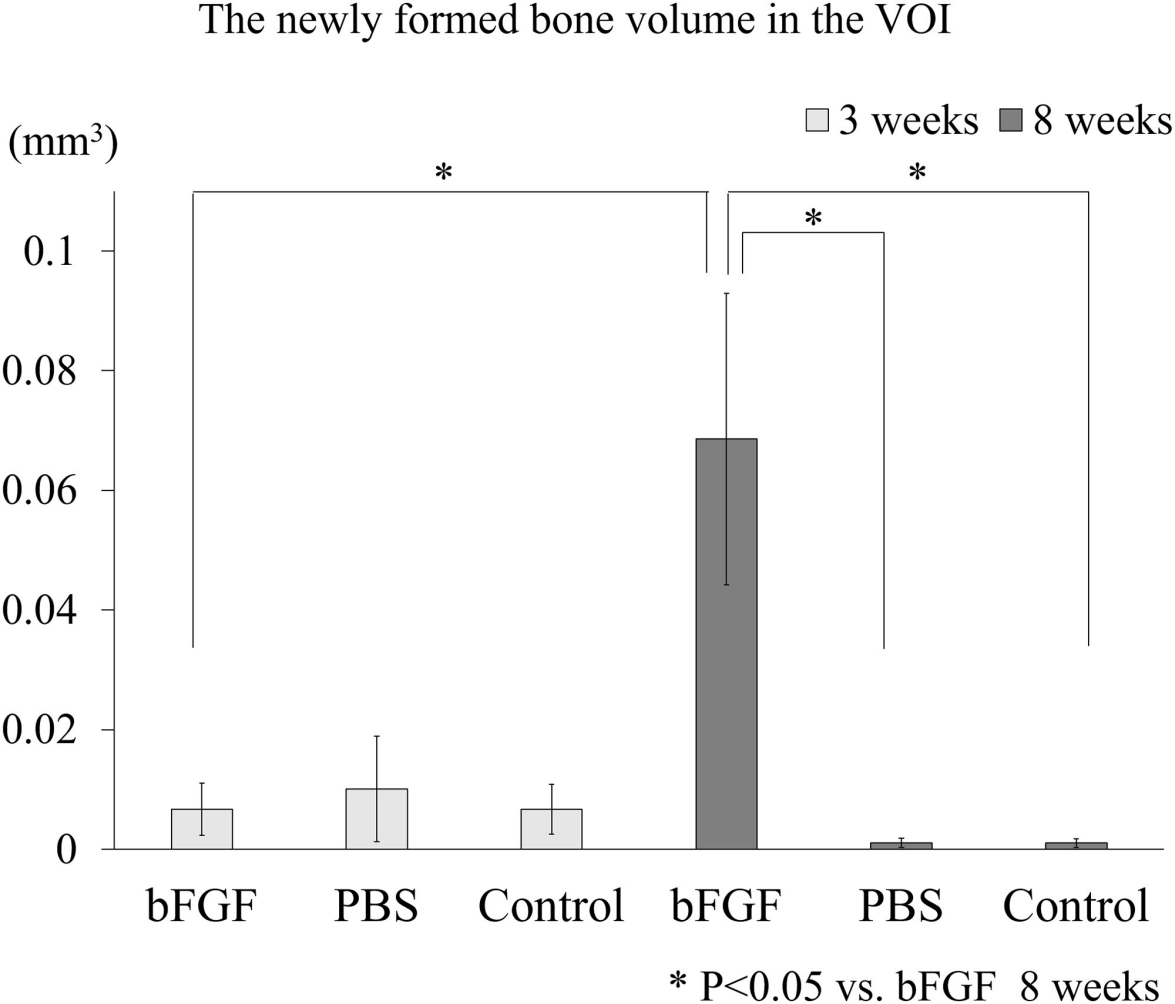
The volume of newly formed bone in the extraction socket. The volume of newly formed bone in the bFGF group at 8 weeks after tooth extraction was significantly greater than the corresponding volumes in the PBS group and the control group at 8 weeks after tooth extraction, as well as in the bFGF group at 3 weeks after tooth extraction. Each error bar denotes 1 standard error.

The volume of the newly formed bone in the alveolar defects was quantified in each group at 3 and 8 weeks after tooth extraction (Fig 5). There was hardly any bone formation (0.0067–0.010 mm^3^) in any of the groups at 3 weeks after tooth extraction, with no significant differences among the groups. On the other hand, the volumes of the newly formed bone at 8 weeks after tooth extraction were 0.0686 mm^3^ (SE 0.0244 mm^3^) in the bFGF group, 0.0010 mm^3^ (SE 0.0008 mm^3^) in the PBS group, and 0.0010 mm^3^ (SE 0.0007 mm^3^) in the control group, showing a significantly greater amount of bone formation in the bFGF group than in the other groups (p = 0.015, against the PBS group; p = 0.015, against the control group). When the volumes of the newly formed bone at 3 and 8 weeks after tooth extraction in the bFGF group were compared, bone formation was found to be significantly greater at 8 weeks (p = 0.015).

### Histological findings

**Fig 6:**
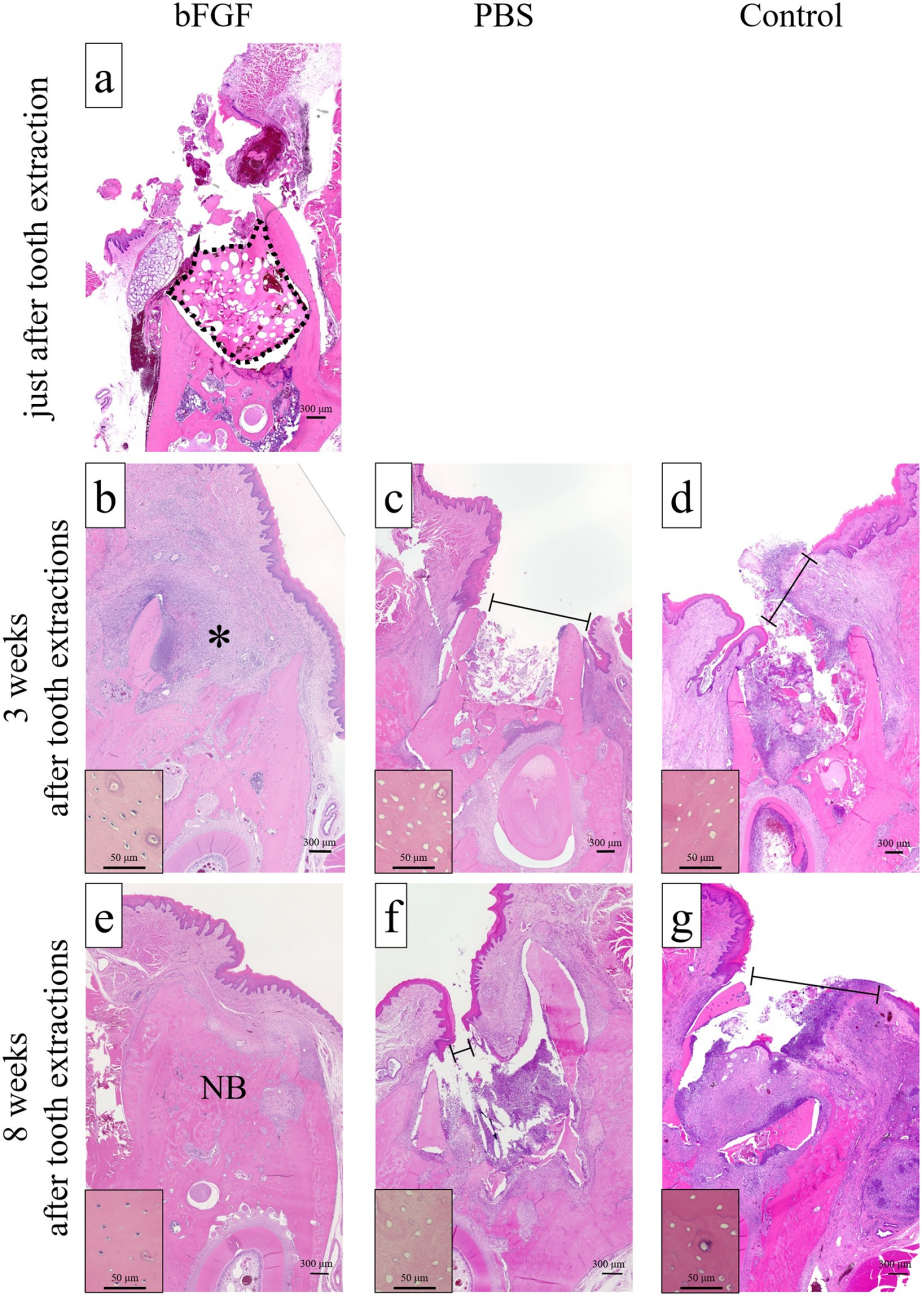
Typical histological findings. (a) Alveolar defect in the bFGF group immediately after tooth extraction. The gelatin hydrogel applied in the defect is indicated as a dotted line. (b) Alveolar defect in the bFGF group at 3 weeks after tooth extraction. (c) Alveolar defect in the PBS group at 3 weeks after tooth extraction. (d) Alveolar defect in the control group at 3 weeks after tooth extraction. (e) Alveolar defect in the bFGF group at 8 weeks after tooth extraction. (f) Alveolar defect in the PBS group at 8 weeks after tooth extraction. (g) Alveolar defect in the control group at 8 weeks after tooth extraction. There was no mucosal disruption in the bFGF group at 3 and 8 weeks after tooth extraction (b, e). The defect was filled with granulation tissue (*) and new bone (NB) at 3 and 8 weeks after tooth extraction, respectively. Numerous osteocytes were presented in their lacunae in the bFGF group at 3 and 8 weeks after tooth extraction (inset b, e). In the group, mild infiltration of inflammatory cells, consisting mainly of neutrophils, was observed with multinucleated giant cells at 3 weeks after tooth extraction, but the infiltration was scarce at 8 weeks after tooth extraction. Mucosal disruption (black line) was present in the PBS group (c, f) and in the control group (d, g) at 3 and 8 weeks after tooth extraction, and there was no bone formation. Numerous empty osteocyte lacunae were observed in the PBS group and in the control group at 3 and 8 weeks after tooth extraction (inset c, d, f, g). Additionally, severe inflammatory cell infiltration, mainly comprising neutrophils, was observed in the PBS group and the control group at 3 and 8 weeks after tooth extraction (hematoxylin and eosin stain; original magnification ×4, inset magnification ×40).

Gelatin hydrogel was present in the extraction sockets of treated rats immediately after tooth extraction (Fig 6a), but did not remain there in any group at 3 and 8 weeks after tooth extraction (Fig 6b–6g). The presence or absence of mucosal disruption in the alveolar defects were evaluated at 3 weeks after tooth extraction (Table 3). In the bFGF group (Fig 6b), mucosal disruption was found in 14.3% (1/7) of alveolar defect samples. In contrast, the corresponding percentages for mucosal disruption in the PBS group (Fig 6c) and the control group (Fig 6d) were 85.7% (6/7) samples and 100% (7/7) samples, respectively, which were significantly higher than in the bFGF group.

**Table 3:** Histologic findings of each group.

	bFGF	PBS	Control
Mucosal disruption (at 3 weeks after tooth extraction)	1/7 (14.3%)	6/7* (85.7%)	7/7* (100%)
Osteonecrosis (at 8 weeks after tooth extraction)	0/7 (0%)	7/7* (100%)	6/7* (85.7%)

*P < 0.05 vs. bFGF

The alveolar defects examined at 8 weeks after tooth extraction showed no osteonecrosis in the bFGF group (Fig 6e). On the other hand, in the PBS group (Fig 6f) and in the control group (Fig 6g), osteonecrosis was present in 100% (7/7) and 85.7% (6/7) of samples, respectively; this was significantly greater than in the bFGF group, similar to the findings at 3 weeks after tooth extraction.

**Fig 7:**
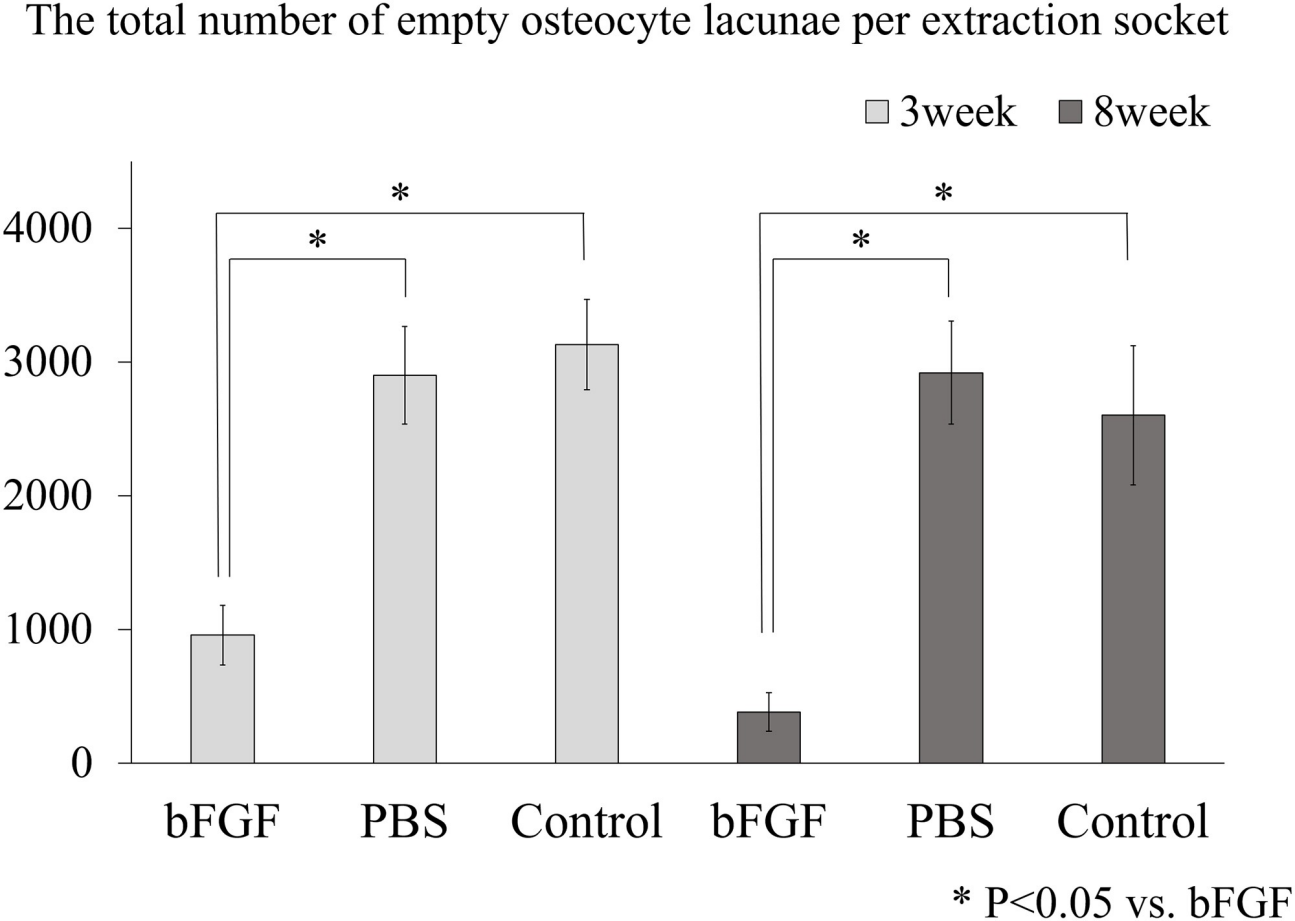
The total number of empty osteocyte lacunae per extraction socket. The total number of empty osteocyte lacunae per extraction socket in the bFGF group at 3 and 8 weeks after tooth extraction was significantly lower than the total empty osteocyte lacunae in the PBS group and the control group at 3 and 8 weeks after tooth extraction. Each error bar denotes 1 standard error.

The total number of empty osteocyte lacunae per extraction socket was quantified in each group at 3 and 8 weeks after tooth extraction (Fig 7). The total number of empty osteocyte lacunae at 3 weeks after tooth extraction were 957 (37.1% of the total cell count, SE 221) in the bFGF group, 2902 (87.2% of the total cell count, SE 364) in the PBS group, and 3130 (87.7% of the total cell count, SE 338) in the control group, showing a significantly lower number in the bFGF group than in the other groups (p = 0.0027, against the PBS group; p = 0.0017, against the control group). In addition, the total number of empty osteocyte lacunae at 8 weeks after tooth extraction were 382 (20.1% of the total cell count, SE 142) in the bFGF group, 2919 (87.7% of the total cell count, SE 385) in the PBS group, and 2602 (73.0% of the total cell count, SE 520) in the control group, showing a significantly lower number in the bFGF group than in the other groups (p = 0.0017, against the PBS group; p = 0.0017, against the control group).

## Discussion

As measures for preventing BRONJ triggered by tooth extraction, infection prophylaxis, including complete removal of potential sources of infection, improvement of oral hygiene, antimicrobial medication before tooth extraction, and closing the extraction socket, have been recommended [5, 18, 19]. However, it is difficult to prevent the occurrence of BRONJ by infection prophylaxis alone, and therefore the development of a new prophylactic measure is awaited. This study was performed to verify whether application of bFGF to the extraction socket would prevent the occurrence of BRONJ after tooth extraction. We performed tooth extraction in rats that were administered BP, and investigated the incidence of osteonecrosis after application of bFGF-containing gelatin hydrogel into the alveolar defects. The incidence of osteonecrosis at 8 weeks after tooth extraction was 0% in the bFGF group, 100% in the PBS group, and 85.7% in the control group, showing a markedly lower rate in the bFGF group than in the other groups. These results suggest that application of bFGF in the extraction socket can prevent the occurrence of BRONJ after tooth extraction. This is an entirely new preventive method that has not been reported previously.

In addition, to examine the effects of bFGF on the oral mucosa, mucosal healing in the extraction socket at 3 weeks after tooth extraction was investigated. The frequency of complete coverage of the extraction socket by mucosal tissue was significantly greater in the bFGF group than in the other groups. It can be inferred from these findings that wound healing in the extraction socket was facilitated by bFGF, and this facilitated the prevention of BRONJ. It can be presumed that inhibition of socket healing due to BP was offset by the growth-stimulating effect of bFGF. These findings suggest that BP-induced inhibition of wound healing may have a significant role in the initiation of BRONJ, as was also observed in a previous study [8].

Although bFGF is clinically used for the treatment of intractable skin ulcers, such as decubitus ulcers, burn ulcers, and diabetic ulcers [9, 20, 21], it is used mainly by repeated administration [22] because bFGF has a short half-life [10]. However, in order to apply it to the extraction socket, a method for achieving a therapeutic effect with a single dose was necessary. To this end, we used a slow-release bFGF using a gelatin hydrogel drug-delivery system. Because bFGF forms an ion complex with gelatin hydrogel, it is released slowly as the gelatin hydrogel degraded enzymatically [12–15, 23]. The time period of gelatin hydrogel degradation in vivo varies, depending on its water content [13–15, 23]. In this study, the water content of the gelatin hydrogel was set at 95%, referring to a previous report [24]. It is demonstrated that gelatin hydrogel with a water content of 95% allowed release of about 20% of incorporated bFGF within the first few hours, and that the remaining bFGF is released slowly as the gelatin hydrogel is degraded over a time period of 2 weeks [14, 24]. The combination ratio of bFGF and gelatin hydrogel was set at 100 μg bFGF/20 mg gelatin hydrogel, based on a previous report [24]. In future, the optimal time profile of bFGF release and the optimal combination ratio to the gelatin hydrogel should be investigated further.

Although this study suggested that bFGF-containing gelatin hydrogel could be an excellent agent to prevent the occurrence of BRONJ after tooth extraction, it is necessary to recognize the limitations of this study. This study did not include a continuous investigation of the same individuals, and therefore, our view that the development of BRONJ was prevented by early mucosal healing in the extraction socket remains speculative. It also remains unclear whether and how factors other than mucosal healing in the extraction socket, facilitated by bFGF, contribute to the inhibition of BRONJ development. It is known that bFGF promotes not only mucosal healing, but also proliferation of osteoblasts, differentiation and migration of osteoclasts, immune function, and angiogenesis [25–31]. These actions are considered to offset the influence of BPs on the jawbone. For instance, even though inhibition of bone growth in the extraction socket by BPs was reported previously [32], growth of bone was observed in this study. It is speculated that not only indirect facilitation of bone growth by early mucosal healing in the extraction socket but also direct actions of bFGF such as improvement of bone remodeling and acceleration of angiogenesis are involved. Elucidation of the mechanism by which bFGF inhibits the occurrence of BRONJ may clarify the pathology of BRONJ, which remains unclear at present.

Currently, clinical research of bFGF-containing gelatin hydrogel for the treatment of avascular necrosis of the femoral head or critical limb ischemia is underway, and a phase II study, which has yielded favorable results, is being completed [33, 34]. We also plan to investigate the usefulness of bFGF-containing gelatin hydrogel in a phase II clinical study in patients who have a history of treatment with BPs.

## Supporting information

S1 Fig Macroscopic, micro-CT, and histological findings of non-zoledronic acid treated rats. Six non-zoledronic acid (ZA)-treated rats underwent unilateral extraction of a lower first molar, in the same manner as ZA-treated rats. Four rats were sacrificed at 3 weeks; the remaining two rats were sacrificed at 8 weeks after tooth extraction. (a) Three weeks after tooth extraction, macroscopic examination showed all non-ZA samples were covered with normal mucosa. (b) All micro-CT images obtained at 3 weeks after tooth extraction showed new bone formation in the extraction sockets, but not at the alveolar crests. (c) Three weeks after tooth extraction, histological examination showed no mucosal disruption in non-ZA samples. (d) Eight weeks after tooth extraction, macroscopic examination showed all non-ZA samples were covered with normal mucosa. (e) All micro-CT images obtained at 8 weeks after tooth extraction showed extraction sockets that were filled with new bone. (f) Eight weeks after tooth extraction, histological examination showed no osteonecrosis in non-ZA samples.
(TIF)

S1 Table Study’s data. (XLSX)
