# Supplementary figures and images for: Prevention of tooth extraction-triggered bisphosphonate-related osteonecrosis of the jaws with basic fibroblast growth factor: An experimental study in rats
Source: PLoS One. 2019 Feb 8;14(2):e0211928. doi: 10.1371/journal.pone.0211928 (PMC6368314; doi:10.1371/journal.pone.0211928)

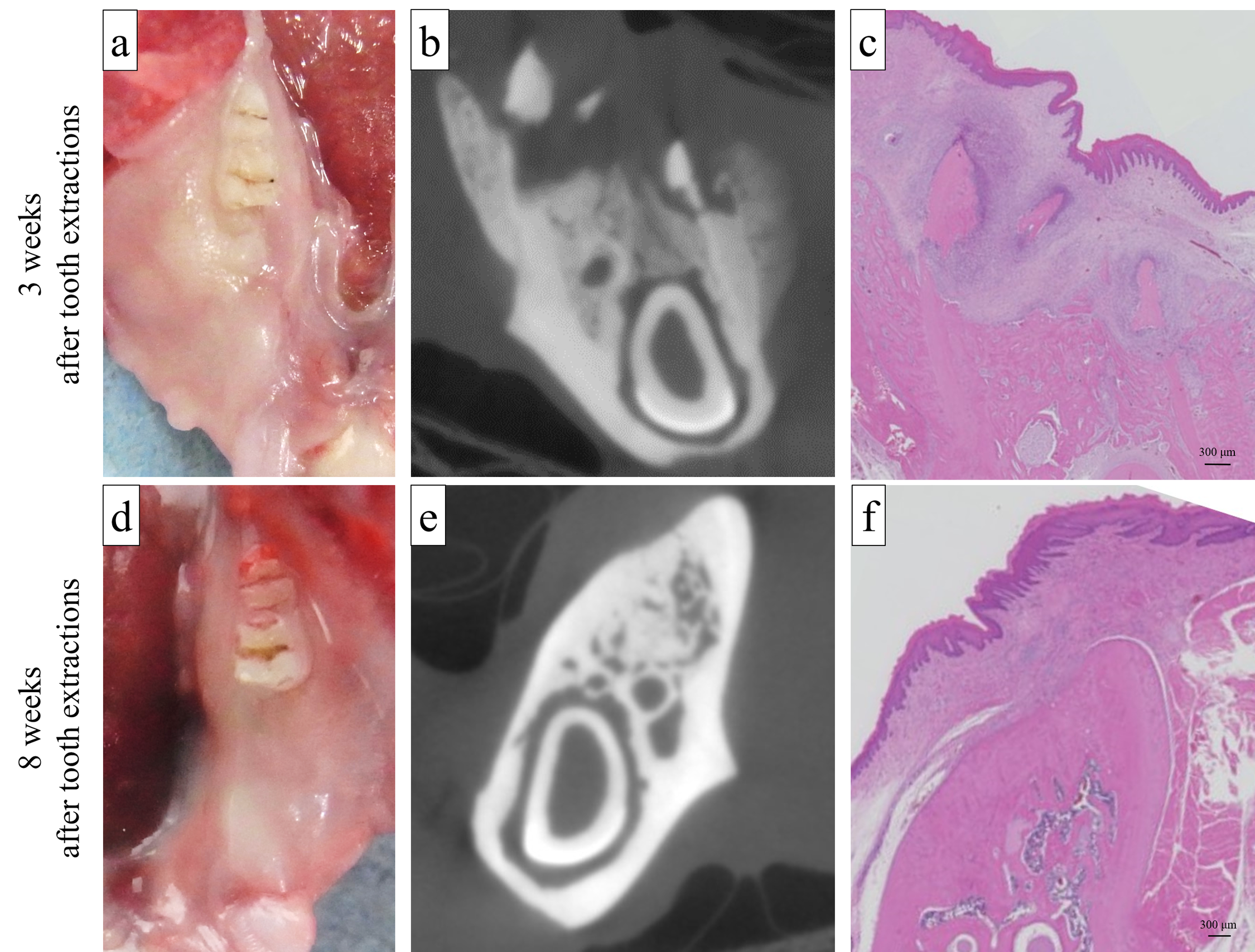

Supplement: S1 Fig — Six non-zoledronic acid (ZA)-treated rats underwent unilateral extraction of a lower first molar, in the same manner as ZA-treated rats. Four rats were sacrificed at 3 weeks; the remaining two rats were sacrificed at 8 weeks after tooth extraction. (a) Three weeks after tooth extraction, macroscopic examination showed all non-ZA samples were covered with normal mucosa. (b) All micro-CT images obtained at 3 weeks after tooth extraction showed new bone formation in the extraction sockets, but not at the alveolar crests. (c) Three weeks after tooth extraction, histological examination showed no mucosal disruption in non-ZA samples. (d) Eight weeks after tooth extraction, macroscopic examination showed all non-ZA samples were covered with normal mucosa. (e) All micro-CT images obtained at 8 weeks after tooth extraction showed extraction sockets that were filled with new bone. (f) Eight weeks after tooth extraction, histological examination showed no osteonecrosis in non-ZA samples. (TIF) [file pone.0211928.s001.tif]
